# miR-29a Modulates GSK3β/SIRT1-Linked Mitochondrial Proteostatic Stress to Ameliorate Mouse Non-Alcoholic Steatohepatitis

**DOI:** 10.3390/ijms21186884

**Published:** 2020-09-19

**Authors:** Ya-Ling Yang, Pei-Wen Wang, Feng-Sheng Wang, Hung-Yu Lin, Ying-Hsien Huang

**Affiliations:** 1 Department of Anesthesiology, Kaohsiung Chang Gung Memorial Hospital and Chang Gung University College of Medicine, Kaohsiung 833, Taiwan; inr453@cgmh.org.tw; 2 Department of Internal Medicine, Kaohsiung Chang Gung Memorial Hospital and Chang Gung University College of Medicine, Kaohsiung 833, Taiwan; wangpw@adm.cgmh.org.tw; 3 Center for Mitochondrial Research and Medicine, Kaohsiung Chang Gung Memorial Hospital, Kaohsiung 833, Taiwan; wangfs@ms33.hinet.net; 4 Core Laboratory for Phenomics & Diagnostics, Department of Medical Research, Kaohsiung Chang Gung Memorial Hospital and Chang Gung University College of Medicine, Kaohsiung 833, Taiwan; 5 Research Assistant Center, Show Chwan Memorial Hospital, Changhua 500, Taiwan; 6 Department of Pediatrics, Kaohsiung Chang Gung Memorial Hospital and Chang Gung University College of Medicine, Kaohsiung 833, Taiwan

**Keywords:** microRNA-29a, NASH, liver fibrosis, GSK3β, SIRT1, proteostatic stress, mitochondrial unfolded protein response

## Abstract

MicroRNA-29a (miR-29a) has been shown to ameliorate hepatocellular damage, such as in the context of non-alcoholic fatty liver disease (NAFLD), steatohepatitis (NASH), and cholestatic injury. However, the mechanism mediating the hepatoprotective effect of miR-29a in diet-induced NASH remains elusive. In the present study, C57BL/6 mice of wild-type (WT) or miR-29a overexpression were fed with methionine–choline sufficient (MCS) or methionine–choline-deficient (MCD) diet for four weeks. The C57BL/6 mice harboring miR-29a overexpression presented reduced plasma AST, hepatic CD36, steatosis, and fibrosis induced by MCD. The TargetScan Release7.2-based bioinformatic analysis, KEGG pathway analysis, and luciferase reporter assay confirmed that miR-29a targets 3′UTR of glycogen synthase kinase 3 beta (*Gsk3b*) mRNA in the HepG2 hepatocyte cell line. Furthermore, miR-29a overexpression in the MCD-fed group resulted in inhibition of *Gsk3b* mRNA and GSK3β protein levels in the liver. GSK3β was notably expressed jointly with the extent of aggregated protein, which was then identified to be associated with mitochondrial unfolded protein response (UPR^mt^), but not with endoplasmic reticulum UPR (UPR^ER^). Additionally, in silico analysis of protein–protein interaction, in vivo, and in vitro correlation analyses of protein expression demonstrated that GSK3β closely associated with sirtuin 1(SIRT1). Finally, the implication of SIRT1-mediated mitochondrial biogenesis in the perturbation of proteostasis was observed. We herein provide novel insight into a hepatoprotective pathway, whereby miR-29a inhibits GSK3β to repress SIRT1-mediated mitochondrial biogenesis, leading to alleviation of mitochondrial proteostatic stress and UPR^mt^ in the context of NASH. miR-29a, GSK3β, and SIRT1 could thus serve as possible therapeutic targets to improve the treatment of NAFLD/NASH.

## 1. Introduction

Non-alcoholic fatty liver disease (NAFLD) is mainly characterized by hepatic steatosis, which arises from the non-alcoholic origin of fat accumulation in the liver. NAFLD is considered a central factor in the pathogenesis of insulin resistance and type 2diabetes [1]. In addition, NAFLD has been shown to be significantly associated with metabolic syndromes, including hypertension and cardiovascular disease, and it is therefore regarded as a clinical manifestation in the complex spectrum of metabolic diseases [2]. Around 20% of NAFLD patients will go on to develop non-alcoholic steatohepatitis (NASH), while approximately 20% of NASH patients will progress to fibrosis, cirrhosis, and finally hepatocellular carcinoma (HCC) as the end-stage [3,4,5]. The manifestation of NASH in humans is evidenced by key histological features such as hepatic steatosis, injury, and fibrosis [6]. 

Multiple hit theory provided an explanation of the pathophysiological mechanisms accounting for NAFLD/NASH [7]. In this model, excessive uptake of free fatty acid into hepatocytes will lead to lipotoxicity [8]. The resulting lipotoxicity primes the liver for “multiple and parallel hit” injuries, such as oxidative stress-induced mitochondrial dysfunction, endoplasmic reticulum (ER) stress, and the activation of pro-inflammatory and fibrogenic pathways [8]. In this regard, mounting evidence has underscored the implication of mitochondria in the activation of inflammatory signaling in the context of NAFLD/NASH [9,10]. Mitochondria are not only key organelles for energy generation and lipid metabolism but also vital signaling platforms for innate immune pathways [11]. Mitochondrial proteostatic stress can induce mitochondrial dysfunction and signal retrograde mitochondria-to-nucleus crosstalk, causing activation of mitochondrial unfolded protein response (UPR^mt^). The signaling of UPR^mt^ necessitates the phosphorylation of eukaryotic translation initiation factor 2 subunit 1 (eIF2α), which subsequently acts to reduce global protein synthesis yet increase the expression of essential transcription factors, including ATF4, ATF5, and C/EBP homologous protein (CHOP). The resulting transcriptional activity boosts critical genes that exert protein refolding or protease activity to attenuate proteostatic stress [11] A growing number of reports have highlighted that UPR^mt^ functions as a nexus mastering the innate immune response [12,13,14]. Furthermore, several reports have been revealed the involvement of proteostatic stress and the emerging role of UPR^mt^ in the progression of NAFLD/NASH [15]. 

MicroRNAs (miRs) are affiliated with a large family of non-coding RNAs. miRs only have approximately 22 nucleotides, yet some of them exert regulatory roles in metabolic homeostasis [16]. By virtue of binding to 3′untranslated region (3′UTR) of target mRNA(s), miRs can act toward mRNA degradation or translational repression [17]. Meanwhile, other regulatory mechanisms have also been discovered, including binding of miRs to 5′UTR of mRNA, mitochondrial transcripts or toll-like receptors [18,19,20]. Recent studies have demonstrated that the miR-29 family plays a critical role in the pathogenesis of liver fibrosis [21]. Of note, miR-29a has been shown to exert protective effects against hepatic steatosis and fibrosis caused by long-term high-fat diet [22]. In addition, enhanced miR-29a expression has been shown to significantly decrease the free cholesterol accumulation in a hepatic steatosis cell model [23]. Our previous studies have demonstrated that miR-29a overexpression in cholestatic mice can significantly inhibit hepatocellular damage and liver fibrosis [24,25,26,27,28]. We further demonstrated that overexpression of miR-29a attenuates fatty acid uptake into the liver and the perturbation of hepatic mitochondrial biogenesis, leading to the prevention of hepatic steatosis, inflammation and fibrosis in the context of obesity-induced NAFLD [29]. In addition, the exogenous administration of a miR-29a mimic leads to hepatic protection and attenuation of mitochondrial proteostatic stress in the cholestatic liver [28]. However, whether miR-29a could exert a neutralizing effect over mitochondrial proteostatic stress on the progression of NAFLD/NASH remains unclear. We herein demonstrate that overexpression of miR-29a ameliorates methionine–choline-deficient (MCD) diet-induced NASH through inhibition of glycogen synthase kinase 3 beta (GSK3β) by binding to its mRNA 3′UTR, leading to restoring imbalanced SIRT1 (sirtuin 1)-mediated mitochondrial biogenesis and proteostatic stress.

## 2. Results

### 2.1. Overexpression of miR-29a Attenuates MCD-Induced Hepatic Steatosis and Fibrosis

**Figure 1:**
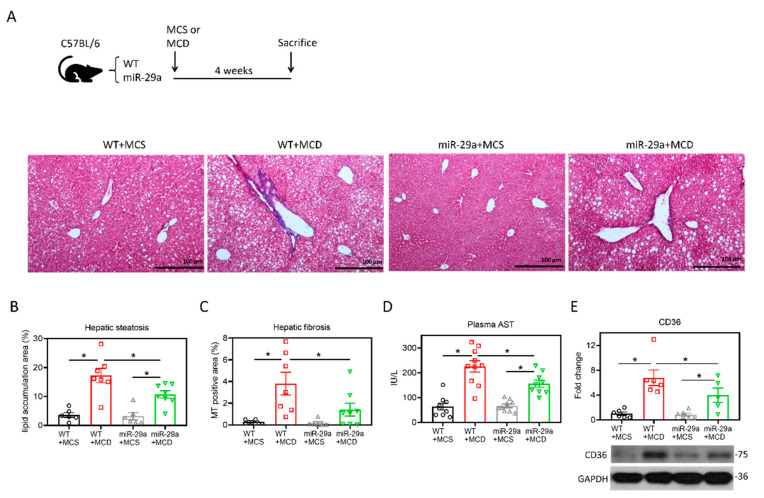
Overexpression of miR-29a attenuates MCD-induced hepatic steatosis and fibrosis. (**A**) Representative images of Masson’s trichrome stain. Vacuole formation and blue stain indicate hepatic lipid accumulation and collagen matrix accumulation, respectively. (**B**,**C**) Quantification of vacuole formation (hepatic steatosis) and blue stain (hepatic fibrosis). (**D**) Plasma aspartate aminotransferase (AST) level was determined with SPOTCHEM EZ automated biochemical analyzer. (**E**) Representative immunoblot result and densitometric quantification of CD36 protein expression. Data from five to ten samples per group are expressed as mean ± SE. *p < 0.05 between indicated groups. MCD, methionine/choline-deficient; MCS, methionine/choline-sufficient.

C57BL/6 mice with wild-type (WT) or overexpression of miR-29a (miR-29a) were fed with methionine–choline sufficient (MCS) or MCD diet for four weeks. We examined the hepatic histological features by Masson’s trichrome staining. As shown in Figure 1A–C, MCD significantly induced hepatic steatosis and fibrosis in the WT group, while this histological alteration was improved in the miR-29a group. The hepatic inflammation indicator aspartate aminotransferase (AST) and positive regulator of hepatic steatosis CD36 [30] corresponded with the histological findings (Figure 1D,E). These results indicate overexpression of miR-29a provides hepatoprotective effects for murine hepatic steatosis, hepatitis, and liver fibrosis.

### 2.2. Target Identification of GSK3β as miR-29a Target in Hepatocyte

**Figure 2:**
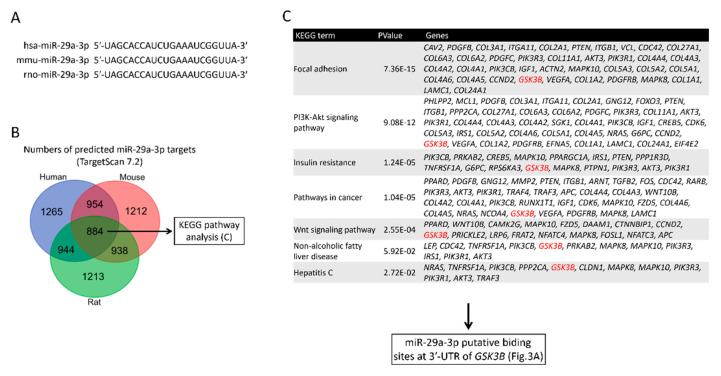
Identification of *GSK3β* as a target gene of miR-29a. (**A**) Alignment analysis of mature miR-29a-3p sequences across species. Note that the sequences are all identical across the three species including, *homo sapiens* (hsa), *Mus musculus* (mmu), and *Rattus norvegicus* (rno). (**B**) Putative gene targets of miR-29a-3p were screened by Targetscan7.2. A Venn diagram illustrates numbers of predicted target mRNAs of hsa-miR-29a-3p, mmu-miR-29a-3p, and rno-miR-29a-3p. (**C**) A KEGG pathway analysis of 884 putative miR-29a-3p target genes in humans. Note that *GSK3B* (highlighted in red) presents in all the pathways and was selected for further assessment.

**Figure 3:**
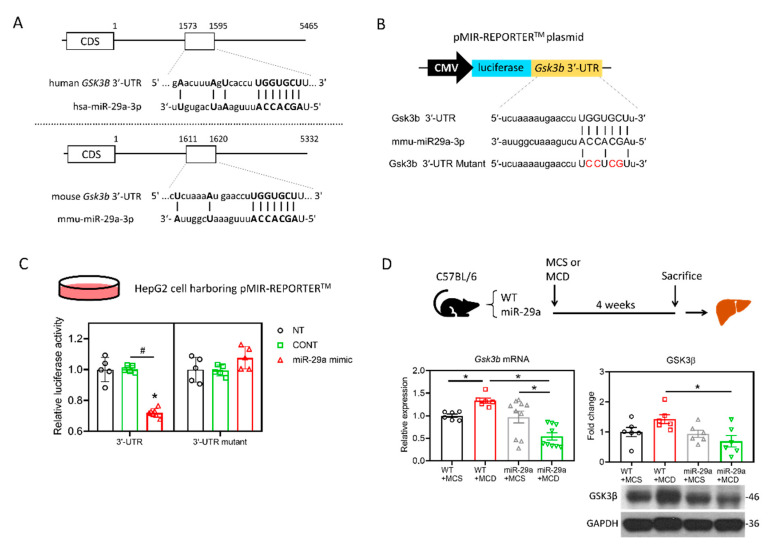
miR-29a suppresses GSK3β expression by direct binding to its 3′UTR. (**A**) Sequence of hsa-miR-29a-3p and its putative binding sites at 3′UTR of human *GSK3B* and mouse *Gsk3b* mRNAs. CDS, coding sequence. Open rectangles represent the region spanning putative miR-29a-3p binding sites. Base-paired nucleotides between 3′UTR and miR-29a-3p are presented in the upper case. (**B**) A schematic representation of the plasmid for cytomegalovirus (CMV)-driven luciferase reporter assay. 3′-UTR of *Gsk3b* or *Gsk3b* mutant was chemically synthesized into the pMIR-REPORT^TM^ luciferase plasmid. Base-paired nucleotides are presented in upper case, while those in red denotes mismatched nucleotides. Plasmid was introduced into human hepatocyte cell line HepG2, followed by transfection of miR-29a mimic, miR negative control or no-treatment. (**C**) Suppression of reporter activities by miR-29a-3p overexpression in HepG2 cells. Cells were no treated (NT) or treated with miR negative control (CONT) or miR-29a mimic for 24h. Five independent experiments for each group were conducted. *p < 0.05 compared with CONT. #*p* < 0.05 between indicated groups. (**D**) *Gsk3b* mRNA level was determined by qRT-PCR. GSK3β protein abundance was measured by Western blot. Data collected from six to ten independent experiments per group are expressed as mean ± SE. * *p* < 0.05 between indicated groups. MCD, methionine/choline-deficient; MCS, methionine/choline-sufficient.

The nucleotide sequences of human miR-29a-3p are identical with those of the mouse and rat, according to the TargetScan database (Figure 2A), indicating a high level of conservation across species. To identify the target mRNAs of hsa-miR-29a-3p, rno-miR-29a-3p, and mmu-miR-29a-3p, we conducted a bioinformatic analysis using the TargetScan Release7.2 database. The analysis revealed a total of 1265, 1212, and 1213 putative target genes that harbor binding sites base-paired with miR-29a-3p in their 3′UTR of humans, mice, and rats, respectively (Figure 2B). The 884 common putative genes of the three species further underwent a KEGG pathway analysis in humans (Figure 2B). We noted that glycogen synthase kinase 3 beta (*GSK3B*) presents in all the seven KEGG pathways (Figure 2C). Given that GSK3β has been reported to promote hepatocellular injury in experimental courses both in vivo [31] and in vitro [32], *GSK3B* was hence selected for further assessment. According to TargetScan Release 7.2, miR-29a-3p presented the identical binding sequence, UGGUGCU, in both humans and mice at positions 1588-1594 and 1613-1619, respectively (Figure 3A). To further validate the complementary relationship of *GSK3B* and miR-29a experimentally, we performed the luciferase reporter assay using human hepatocyte cell line HepG2. The sequence of *Gsk3b* 3′UTR or *Gsk3b* 3′UTR mutant were cloned into pMIR-REPORTER^TM^ plasmid’s multiple cloning site after cytomegalovirus (CMV)-driven luciferase (Figure 3B). Plasmid was then introduced into HepG2 cells, followed by treatment with miR negative control sequence (CONT), miR-29a mimic, or no treatment (NT). In cells with *Gsk3b* 3′UTR, transduction of miR-29a mimic significantly decreased the luciferase reporter activity, whereas cells with *Gsk3b* 3′UTR mutant treated with miR-29a mimic presented no effect on the luciferase signal (Figure 3C). These results confirmed that *GSK3B* is a target of miR-29a in hepatocytes. We then observed the GSK3β profiling in the mouse liver. As shown in Figure 3D, *Gsk3b* mRNA level in the WT group was increased by MCD, while that in the miR-29a group was decreased. The miR-29a+MCD group exhibited a lower level of *Gsk3b* mRNA than that of the WT+MCD group. Similarly, the miR-29a+MCD group exhibited a decreased GSK3β protein level compared with the WT+MCD group (Figure 3D). These data indicate that binding of miR-29a on *GSK3β* 3′UTR accounts for the attenuation of GSK3β, whereby the GSK3β abundance is inhibited by miR-29a in the liver under MCD stress.

### 2.3. miR-29a/GSK3β Axis Alleviates Mitochondrial Proteostasic Stress via Restoring Perturbation of SIRT1-Mediated Mitochondrial Biogenesis

**Figure 4:**
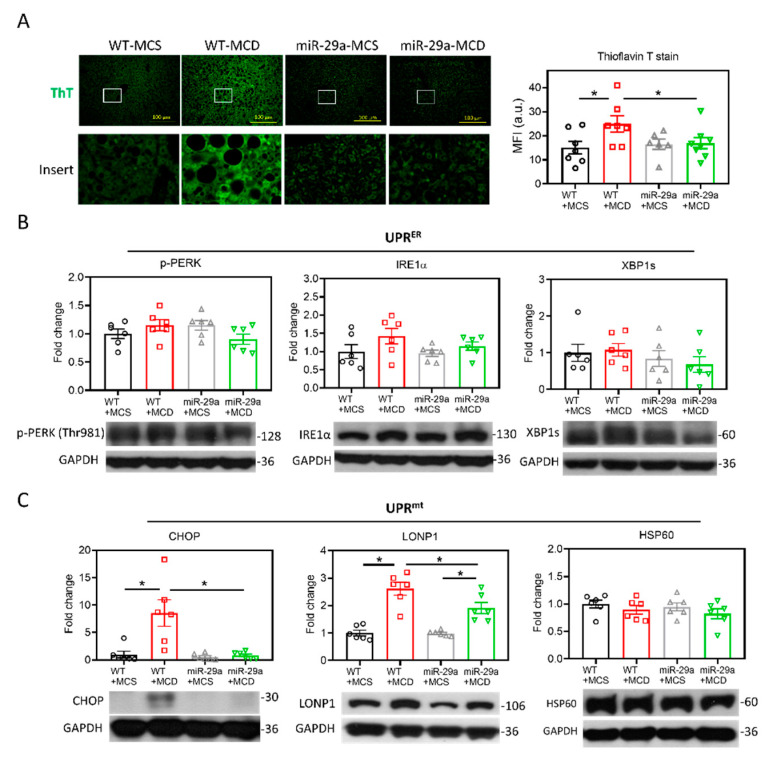
Overexpression of miR-29a alleviated mitochondrial proteostasis stress. (**A**) Misfolded protein aggregation in live tissue was stained by thioflavin T (ThT). White open inserts in the upper panel are enlarged in the lower panel. Scale bar, 100 μm. Mean fluorescence intensity (MFI) of ThT was determined using Image J. N = 6–8 in each group. a.u., arbitrary unit. (**B**) Representative blots and densitometric results of p-PERK, IRE1α and XBP1s, indicators of unfolded protein response of the endoplasmic reticulum (UPR^ER^). N = 6 in each group. (**C**) Representative blots and densitometric results of CHOP, LONP1, and HSP60, indicators of unfolded protein response of the mitochondria (UPR^mt^). N = 6 in each group. Data are expressed as mean ± SE. * *p* < 0.05 between indicated groups. MCD, methionine/choline-deficient; MCS, methionine/choline-sufficient.

As GSK3β has been shown to play a positive factor in the UPR of injured livers [31], we examined proteostatic stress by detecting the aggregated protein as stained by thioflavin T (ThT), which serves as a ligand for imaging protein aggregates expressing cross-β sheet structure [33]. As shown in Figure 4A, the MCD diet induced an increase in the ThT signal in the liver of the WT group, whereas MCD had no impact on that of the miR-29a group. Notably, the miR-29a+MCD group showed a lower ThT signal than the WT+MCD group (Figure 4A), indicating that overexpression of miR-29a effectively ameliorates proteostatic stress. To clarify the involvement of endoplasmic reticulum (ER) or mitochondria in this proteostatic stress, we examined the indicators for UPR^ER^ and UPR^mt^ by measuring phosphorylated protein kinase-like endoplasmic reticulum kinase (p-PERK), inositol-requiring enzyme-1α (IRE1α), spliced X-box binding protein 1 (XBP1s), CHOP, lon peptidase 1 (LONP1), and heat shock protein 60 (HSP60). As shown in Figure 4B, the UPR^ER^ panel in the liver showed no significant alteration by either MCD diet or the miR-29a overexpression. Additionally, no alteration was observed in the HSP60 level of the UPR^mt^ panel (Figure 4C). Of note, the WT+MCD group had higher CHOP and LONP1 levels than the WT+MCS group; while the miR-29a+MCD group showed higher LONP1 than the miR-29a+MCS group. Furthermore, the miR-29a+MCD group exhibited reduced CHOP and LONP1 levels compared with the WT+MCD group. These results indicate that miR-29a-alleviation of proteostatic stress is associated with UPR^mt^.

**Figure 5:**
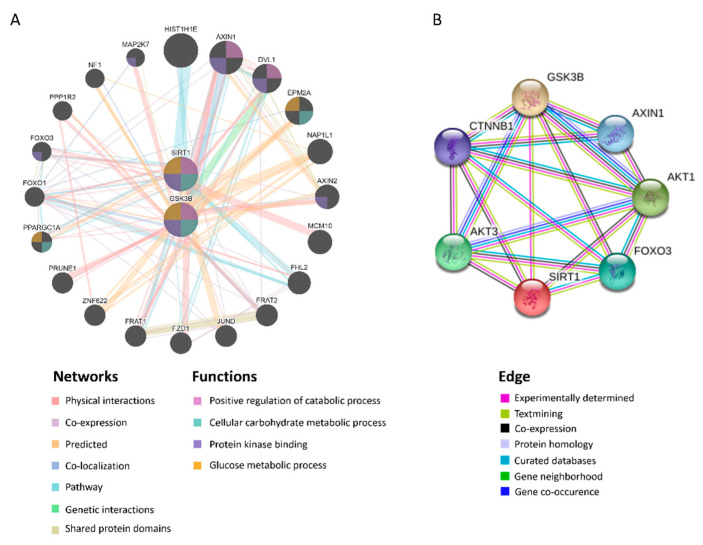
GSK3β is closely associated with SIRT1. Predicted protein–protein interactions (PPI) between GSK3β and SIRT1 using STRING and GeneMANIA servers. (**A**) Predicted functional partners are shown after considering physical interaction (67.64%), co-expression (13.5%), predicted (6.35%), co-localization (6.17%), pathways (4.35%), genetic interactions (1.40%), and shared protein domains (0.59%). The mutual functions of GSK3β and SIRT1 include positive regulation of the catabolic process, cellular carbohydrate metabolic process, protein kinase binding, glucose metabolic process. (**B**) The edges are determined by experimentally determined, textmining, co-expression, protein homology, curated databases, gene neighborhood, gene co-occurrence.

**Figure 6:**
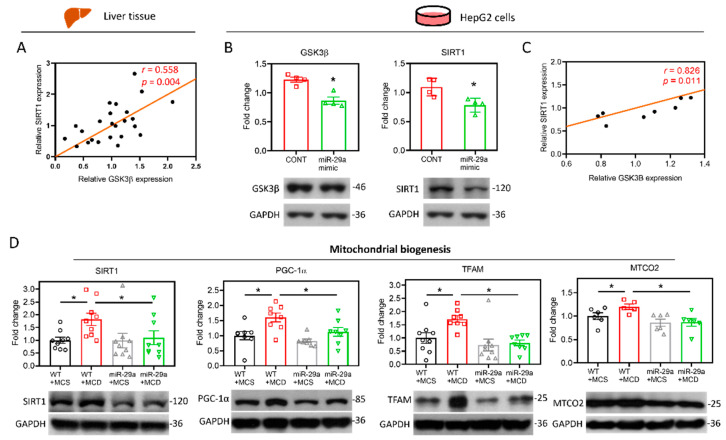
miR-29a/GSK3β axis counterbalances the perturbation of SIRT1-mediated mitochondrial biogenesis. (**A**) Pearson’s correlation of GSK3β with SIRT1 in the mouse liver (N = 24). (**B**) Human HepG2 cells were transfected with miR negative control (CONT) or miR-29a mimic for 24h. The protein expression level of GSK3β and SIRT1 was determined by Western blot. GAPDH level as loading control (N = 4). (**C**) Pearson’s correlation of GSK3β with SIRT1 in the HepG2 cells (N = 8). *p < 0.05 compared with CONT group. (**D**) Representative blots and densitometric results of SIRT1, PGC-1α, TFAM and MTCO2, which represent indicators of mitochondrial biogenesis. N = 6–9 in each group.

The induction of UPR^mt^ can be activated by SIRT1-mediated mitochondrial biogenesis, which triggers the protein folding workload in mitochondria [34]. In addition, SIRT1 has been shown to directly interact with GSK3β [35], and has been identified to be mandatory for GSK3β signaling in promoting hepatocellular injury [32]. In tandem with those observations, in silico protein–protein interactions (PPI), and analyses on STRING and GeneMANIA servers revealed the correlation between GSK3β and SIRT1 (Figure 5A,B). The gene set rich in GSK3β and SIRT1 is responsible for positive regulation of the catabolic process, cellular carbohydrate metabolic process, protein kinase binding, and glucose metabolic process (Figure 5B). Moreover, we examined the strength of the relationship between the GSK3β protein expression level and that of SIRT1 in vivo, wherein we noted that hepatic GSK3β presented a positive correlation with SIRT1 (*r* = 0.558, *p* < 0.05. Figure 6A). The in vitro cell culture system using HepG2 cells demonstrated that transfection of the miR-29a mimic reduced both GSK3β and SIRT1 protein expression levels in comparison to miR negative control-transfected group (Figure 6B). In addition, GSK3β was positively correlated with SIRT1 in HepG2 cells (*r* = 0.826, *p* < 0.05. Figure 6C). We, therefore, hypothesized that the mechanism of miR-29a/GSK3β signaling modulation of mitochondrial proteostasis may lie in SIRT1-mediated mitochondrial biogenesis. To determine this, we observed the canonical biogenesis pathway SIRT1/PPARG coactivator 1 alpha (PGC-1α)/mitochondrial transcription factor A (TFAM), as well as mitochondrial cytochrome c oxidase subunit 2 (MTCO2). As shown in the mitochondrial biogenesis panel, MCD increased the level of SIRT1/PGC-1α/TFAM/MTCO2 in the WT group, while induction was significantly suppressed in the miR-29a group (Figure 6D). Collectively, these results suggest that the miR-29a/GSK3β axis alleviates mitochondrial proteostatic stress via restoring perturbation of SIRT1-mediated mitochondrial biogenesis.

## 3. Discussion

**Figure 7:**
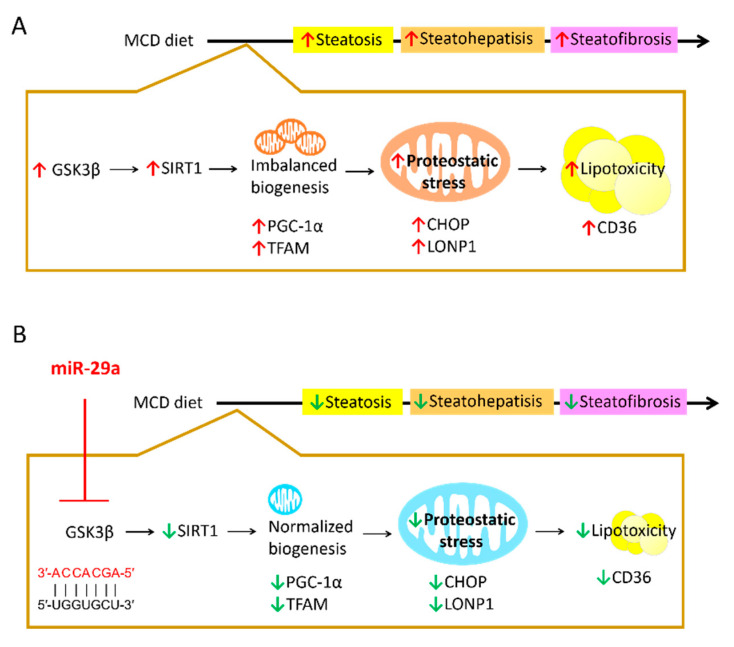
Proposed model of the miR-29a-relayed pathway in the prevention of diet-induced hepatic injury. (**A**) MCD diet leads to an increase in GSK3β, which induces imbalanced mitochondrial biogenesis mediated by SIRT1. As such, protein folding workload in mitochondria can stimulate proteostatic stress, leading to mitochondrial malfunction and subsequent hepatic lipid accumulation. Increased hepatic lipotoxicity can contribute to the progression of steatosis, steatohepatitis and steatofibrosis. (**B**) miR-29a can suppress GSK3β expression by direct binding to its 3′UTR to reverse the aberrant SIRT1-mediated mitochondrial biogenesis and mitochondrial proteostatic stress, ultimately mitigating the pathological progression. Red arrow: increase; Green arrow: decrease.

In this study, we demonstrated that miR-29a exerts a hepatoprotective effect by inhibiting GSK3β through binding to its mRNA 3′UTR to suppress imbalanced mitochondria biogenesis mediated by SIRT1, subsequently leading to amelioration of proteostatic stress and UPR^mt^. The proposed model depicting the mechanism underlying miR-29a/GSK3β/SIRT1 axis is illustrated in Figure 7.

Although mounting evidence indicates the emerging role of miR-29a on preventing the progression of NAFLD/NASH [22], its molecular underpinnings in modulating lipotoxicity in hepatocytes remain unclear. An MCD diet has been shown to cause impaired mitochondrial β-oxidation, leading to hepatic steatosis [36]. The contribution of dysregulation of mitochondrial bioenergetics, structural integrity and dynamics, and mitochondrial DNA damage to NASH have also been noted [37]. CD36, which functions as a fatty acid translocase and a negative regulator of lipid accumulation, links lipotoxicity to mitochondrial homeostasis in hepatocytes [38]. Our previous study showed that overexpression of miR-29a provides resistance against NASH induced by long term high-fat-diet by suppressing the expression of CD36 as well as restoring mitochondrial biogenesis [29]. In the present study, we demonstrated that MCD-induced hepatic CD36 level and the presence of steatosis can be restored in the context of miR-29a overexpression, supporting miR-29a’s role in counteracting lipid accumulation.

Inhibition of GSK3β has been reported as an effective hepatoprotective approach for liver injury induced by ischemia-reperfusion [39] as well as for acute liver failure (ALF) induced by D-galactosamine and lipopolysaccharide (D-GalN/LPS) [31]. In this study, we identified that miR-29a acts to directly bind to 3′UTR of *Gsk3b* mRNA, leading to its inhibition in the hepatocytes. In addition, the reduced expression level of GSK3β in the liver presents concurrently with the amelioration of hepatic steatosis and fibrosis, indicating the miR-29a/GSK3β pathway acts significantly in the hepatoprotective effect.

SIRT1, which is a master regulator of mitochondrial biogenesis and lipid metabolism [40], has been reported to interact with GSK3β [35]. Additionally, SIRT1 has been shown necessary for promoting oxidative stress-induced MEF cell death [41]. Indeed, Koga et al. has identified that the up-regulation of SIRT1 contributes to tunicamycin-induced hepatic injury, while the expression of SIRT1 can be mediated by GSK3β [32]. In this study, the expression manner of SIRT1 manifested in line with that of GSK3β, supporting the notion that the GSK3β/SIRT1 axis plays a role in the pathophysiology of NASH and that this axis can be suppressed by miR-29a to exert hepatoprotection. 

In mammalian cells and *C. elegans*, SIRT1-mediated biogenesis can induce a mito-nuclear protein imbalance, resulting in increased protein folding workload in mitochondria via the activation of UPR^mt^ [34,42]. Our previous study reported that intravenous injection of miR-29a mimic can mitigate bile duct ligation-induced UPR^mt^ in the liver, as evidenced by decreases in the protein levels of CHOP, LONP1, and HSP60 [28]. In this study, we further demonstrated overexpression of miR-29a leads to reduced accumulation of misfolded protein induced by MCD. This proteostatic stress is predominantly associated with UPR^mt^ indicators (CHOP and LONP1), but not UPR^ER^. Interestingly, the UPR^mt^ chaperonin HSP60 appeared not to be implicated, indicating that proteolytic activity executed by the mitochondrial protease system may be a dominant mechanism in response to NASH-induced proteostatic stress, compared with protein refolding activity maintained by the chaperonin system. Nevertheless, further study is warranted to elucidate the intricate protein quality control mechanism. 

The manner in which SIRT1 expression level can be regulated by GSK3β has been demonstrated by the presence of direct physical interaction between GSK3β and SIRT1 in adipocytes [35] and the essential role of GSK3β in relaying SIRT1 signaling in promoting hepatocellular injury [32]. In this study, PPI analysis in silico shows that physical interaction is the primary relationship between GSK3β and SIRT1. In contrast, Nan et al. has demonstrated that SIRT1 expression level can be regulated by direct binding of miR-29a on its 3′UTR in cervical cancer cells [43]. As such, whether SIRT1 expression targeted by miR-29a underpins the molecular mechanism in hepatocytes and liver of NASH model requires further study for clarification.

In summary, this investigation provides novel insight into a hepatoprotective pathway whereby miR-29a inhibits GSK3β to repress SIRT1-mediated mitochondrial biogenesis, leading to alleviation of mitochondrial proteostatic stress and UPR^mt^ in the context of MCD diet-induced NASH. Our results indicate that miR-29a could be a promising therapeutic target for NAFLD/NASH. Moreover, the inhibition of GSK3β, SIRT1 activity, and proteotoxicity could concomitantly serve as a viable therapeutic approach to enhance NAFLD/NASH treatments.

## 4. Materials and Methods

### 4.1. Ethics Statement

The animal protocol was reviewed and approved by the Institutional Animal Care and Use Committee (IACUC) of Chang Gung Memorial Hospital (Approval number: 2018092004; Approval day: 12 07 2019). C57BL/6 mice weighing 25–35 g were purchased from a standardized SPF Laboratory (BioLASCO, Taipei, Taiwan). All animals were housed at the Center for Laboratory Animals of Kaohsiung Chang Gung Memorial Hospital in compliance with the Institutional Animal Care and Use Committee guidelines and were individually housed under controlled temperature (22 °C), humidity (55%), and lighting (12:12-h light-dark cycle), with free access to water and food.

### 4.2. Construction and Breeding of the miR-29a Transgenic Mouse Colony

Transgenic mice harboring overexpression of miR-29a driven by the phosphoglycerate kinase 1 (PGK1) promoter were bred and housed in a specific pathogen-free rodent barrier, as previously described [29]. Briefly, human miR-29a precursor full-length cDNA under the control of human phosphoglycerate kinase 1 (PGK-1) promoter was constructed using PCR protocol. Bioinformatic data reveal high homology between mouse and human miR-29a and high similarity of their putative targets [44]. The genotyping of the mice was examined by PCR method in accordance with previously established protocol [45]. miR-29aTg mice were obtained from littermates that presented positive genotyping results. The miR-29a expression level in the liver of two mouse lines, WT and miR-29aTg, was confirmed by qPCR (Supplementary Figure S1). The appearance of the WT and miR-29Tg mouse groups showed no difference. As previously described, the WT and miR-29Tg mouse groups fed a chow diet presented similar phenotype characteristics, including body weight, physical activity, and liver weight [29].

### 4.3. Animal Model and Experimental Protocol

Six to ten mice in each group were used for all experiments. The mice were categorized into either the “MCS diet” group or the “MCD diet” group. MSD and MCD diet (A02082003B and A02082002B), respectively. OpenSource Diet was purchased from Research Diets, Inc. Eight-week-old male mice underwent a four-week feeding course. The bodyweight of the mice was recorded on a daily basis. In the course of sacrifice, liver tissues were stored at −80 °C until the execution of laboratory processes and assays.

### 4.4. Masson’s Trichrome Stain

For histological examination, liver tissues were preserved in 10% formaldehyde, embedded in paraffin, and cut into 2-μm thick sections and underwent Masson’s trichrome staining. The hepatic steatosis and fibrosis were determined by quantification of the lipid droplets area and blue-stained area, respectively. Images were photographed for ten low-power fields per slide (magnification, 40×), as previously described [46].

### 4.5. Blood Biochemical Analysis

Plasma was preserved at -80 °C until the execution of biochemical analysis. The plasma aspartate aminotransferase (AST) level of the mice was analyzed with a SPOTCHEM EZ (SP-4430, Arkray, KYO, JPN) automated biochemical analyzer.

### 4.6. In Silico miR-29-Targeted mRNA Interaction Analysis

Putative target mRNAs of hsa-miR-29a-3p, mmu-miR-29a-3p, and rno-miR-29a-3p were analyzed using the TargetScan release 7.2 database (http://www.targetscan.org/vert_72/) [47]. The raised genes that had a putative binding site of miR-29a-3p of the human, mouse, and rat at the 3′UTR were inputted into http://bioinformatics.psb.ugent.be/webtools/Venn/for the Venn diagram. The resultant 884 common putative genes across the three species were subjected to the DAVID Bioinformatics Resources 6.8 (https://david.ncifcrf.gov/tools.jsp) to list the Kyoto Encyclopedia of Genes and Genomes (KEGG) pathways in humans. 

### 4.7. In Silico Analysis of Protein–Protein Interaction (PPI)

Both GeneMANIA and STRING servers were used for analyzing PPI. GeneMANIA is an open website for building PPI networks and predicting gene function [48]. This website analyzes genes or gene lists through bioinformatics methodologies, including physical interaction, co-expression, co-location, enrichment analysis, and prediction. STRING is an online data mining platform for the determination of PPI, including physical and functional interplays [49].

### 4.8. Luciferase Reporter Assay

The sequence of *Gsk3b* 3′UTR or *Gsk3b* 3′UTR mutant were cloned into pMIR-REPORTER^TM^ plasmid’s multiple cloning site after CMV-driven luciferase (Figure 3B). To observe luciferase signal, the resultant reporter plasmid was then introduced into the HepG2 hepatocellular cell line, followed by treatment with miR negative control sequence (CONT), miR-29a mimic, or no treatment (NT). Firstly, HepG2 cells were seeded at 1.8 × 10^6^ cells in a 100 mm dish for 18 h. Secondly, 6 μg reporter plasmids with *Gsk3b* 3′UTR or *Gsk3b* 3′UTR mutant were individually transfected into a 100 mm dish cultivating HepG2 cells in the presence of Turbofect transfection reagent (#R0531, Thermo Fisher Scientific, Rockford, IL, USA) for 20 h. Thirdly, HepG2 were trypsinized and seeded at 9 × 10^5^ cells in a 60 mm dish with fresh growth medium. Fourthly, 25 nM miR-29a-3p mimic (C-300504-07-0050, GE Healthcare Dharmacon, IN, USA), miRs negative control (CN-001000-01-50, GE Healthcare Dharmacon), or no-treatment (NT) were transfected into cells in the presence of RNAiMAX transfection reagent (#13778-150, Invitrogen, Carlsbad, CA, USA) for 24 h. Finally, cells were lysed for the detection of luciferase signal with Neolite Reporter Gene Assay System (PerkinElmer, Waltham, MA, USA).

### 4.9. Quantitative Real-Time PCR (qPCR)

Total RNA was extracted by using TRIzol^®^ reagent (15596026, Invitrogen, Carlsbad, CA, USA) from the liver tissue and then underwent reverse transcription to yield cDNA with an oligodeoxynucleotide primer (oligo dT15)-based method according to the manufacturer’s protocol (M1701, Promega, Madison, WI, USA). The qPCR reaction for *Gsk3b*, and normalization control *beta-actin* was conducted with 2× SYBR Green PCR Master Mix (04887352001, Roche Molecular Systems, Inc., Pleasanton, CA, USA) on LightCycler480^®^ (Roche). Each PCR reaction included 0.5 μM forward and reverse primers, 30 ng of cDNA, and 1× SYBR Green PCR Master Mix in a total reaction volume of 10 μL. The qPCR program included an initial denaturation step at 95 ℃ for 10 min, followed by 45 cycles of denaturation at 95 ℃ for 30 s, annealing at 62 ℃ for 15 s, and extension at 72 ℃ for 25 s, with a final step for melting curve analysis. The primers sequence was as follows: *Gsk3b* forward 5′- GAGCTGATGACTAGGGCTGT -3′, *Gsk3b* reverse 5′- GTATA AGGGCCGCCAAGAGA -3′; *beta-actin* forward 5′-CAGCCTTCCTTCTTGGGTATG-3′, *beta-actin* reverse sequence 5′-GGCATAGAGGTCTTTACGGATG-3′. The calculation of relative gene expression was based on the comparative cycle threshold (CT) method, whereby the value of the target gene was given by 2^−(△CT target−△CT calibrator)^ or 2^−△△^*^C^*^T^. 

### 4.10. Western Blotting

15 mg of liver tissue or 1 × 10^6^ cells were lysed in protein lysis buffer (#17081, iNtRON Biotechnology), homogenized and then centrifugated. The resulting supernatant lysates underwent protein quantitation measurement using Bio-RAD protein assay, in accordance with the manufacturer’s protocol (LIT33, Bio-RAD). The bovine serum albumin (BSA) was used as a standard to construct a standard curve for the relative quantitation of the proteins in the samples. A total of 40 µg proteins extracted from the liver underwent standard Western blot courses, including separation by SDS-PAGE and transfer onto PVDF membrane, which was then incubated with primary antibodies at 4 °C overnight. Primary antibodies were listed as follows: CD36 (1: 5000; NB400-144, NOVUS, CO, USA), CHOP (1: 1000; #2895, Cell Signaling, MA, USA), GSK3β (1: 5000; #9315, cell signaling, MA, USA), HSP60 (1: 5000; sc-1052, Santa Cruz, CA, USA), IRE1α (1: 4000, #3294, Cell Signaling, MA, USA), MTCO2 (1: 20,000, 550701-1-AP, Proteintech, IL, USA), LONP1 (1: 2000; 15440-1-AP, Proteintech, IL, USA), TFAM (1: 5000; sc-166965, Santa Cruz, CA, USA), PGC-1α (1: 5000; GTX31921, genetex, CA, USA), SIRT1 (1: 5000, 13161-1-AP, Proteintech, IL, USA), XBP1s (1: 1000,#12782, Cell Signaling, MA, USA), p-PERK(1: 2000, sc-32577, Santa Cruz, CA, USA), and glyceraldehyde 3-phosphate dehydrogenase (GAPDH, 1: 100,000; 60004-1-lg, Proteintech, IL, USA). Following two washes, horseradish peroxidase-conjugated secondary antibodies such as horseradish peroxidase-coupled anti-rabbit immunoglobulin-G antibodies (1: 5000; NEF812001EA, PerkinElmer, MA, USA) or HRP anti-mouse immunoglobulin-G antibodies (1: 10,000; NEF822001, PerkinElmer) was used to generate a chemiluminescent signal. The incubation condition was set at room temperature for 1h. The blots were developed with an ECL Western blotting system and exposed to film (GE28-9068-37, GE Healthcare, IL, USA). The signals were quantified using Quantity One^®^ 1-D analysis software (Bio-Rad Laboratories). The accurate quantitative value of the target protein was normalized by its corresponding GAPDH.

### 4.11. Statistical Analysis

All values are expressed as mean ± standard error (SE). Differences between the two datasets were evaluated by two-tailed unpaired Student’s t-tests. Statistical tests between multiple datasets were analyzed using a one-way analysis of variance (ANOVA) followed by post hoc least significant difference (LSD) test. A p-value under 0.05 was considered statistically significant.

## Supplementary Materials

Supplementary materials can be found at https://www.mdpi.com/1422-0067/21/18/6884/s1, Figure S1: miR-29a expression level in WT and miR-29aTg groups.
