# Supplementary material for: miR-29a Modulates GSK3β/SIRT1-Linked Mitochondrial Proteostatic Stress to Ameliorate Mouse Non-Alcoholic Steatohepatitis
Source: Int J Mol Sci. 2020 Sep 19;21(18):6884. doi: 10.3390/ijms21186884 (PMC7555728; doi:10.3390/ijms21186884)
Supplement: Supplementary file 1 [file ijms-21-06884-s001.pdf]

## Supplementary materials

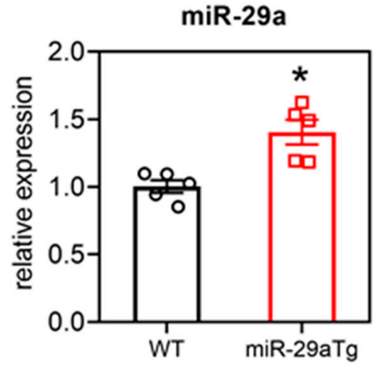


**Supplementary Figure S1. miR-29a expression level in WT and miR-29aTg groups.** Liver tissue of two mouse lines WT and miR-29aTg (N=5 each group) was collected for the detection of miR-29a expression level by qPCR. sno202 level served as normalization control. *$p<0.05$ between WT and miR-29aTg.
